# Allosteric Inhibitory Molecular Recognition of a Photochromic Dye by a Digestive Enzyme: Dihydroindolizine makes α-chymotrypsin Photo-responsive

**DOI:** 10.1038/srep34399

**Published:** 2016-09-28

**Authors:** Damayanti Bagchi, Abhijit Ghosh, Priya Singh, Shreyasi Dutta, Nabarun Polley, Ismail.I. Althagafi, Rabab S. Jassas, Saleh A. Ahmed, Samir Kumar Pal

**Affiliations:** 1 Department of Chemical, Biological and Macromolecular Sciences, S. N. Bose National Centre for Basic Sciences, Block JD, Sector III, Salt Lake, Kolkata 700 106, India; 2 Computer Service Cell, S. N. Bose National Centre for Basic Sciences, Block JD, Sector III, Salt Lake, Kolkata 700 106, India; 3 Department of Chemistry, Faculty of Applied Sciences, Umm Al-Qura University, 21955 Makkah, Saudi Arabia; 4 Chemistry Department, Faculty of Science, Assiut University, 71516 Assiut, Egypt

## Abstract

The structural-functional regulation of enzymes by the administration of an external stimulus such as light could create photo-switches that exhibit unique biotechnological applications. However, molecular recognition of small ligands is a central phenomenon involved in all biological processes. We demonstrate herein that the molecular recognition of a photochromic ligand, dihydroindolizine (DHI), by serine protease α-chymotrypsin (CHT) leads to the photo-control of enzymatic activity. We synthesized and optically characterized the photochromic DHI. Light-induced reversible pyrroline ring opening and a consequent thermal back reaction *via* 1,5-electrocyclization are responsible for the photochromic behavior. Furthermore, DHI inhibits the enzymatic activity of CHT in a photo-controlled manner. Simultaneous binding of the well-known inhibitors 4-nitrophenyl anthranilate (NPA) or proflavin (PF) in the presence of DHI displays spectral overlap between the emission of CHT-NPA or CHT-PF with the respective absorption of *cis* or *trans* DHI. The results suggest an opportunity to explore the binding site of DHI using Förster resonance energy transfer (FRET). Moreover, to more specifically evaluate the DHI binding interactions, we employed molecular docking calculations, which suggested binding near the hydrophobic site of Cys-1-Cys-122 residues. Variations in the electrostatic interactions of the two conformers of DHI adopt unfavorable conformations, leading to the allosteric inhibition of enzymatic activity.

All crucial functions of living organisms are mediated by complex interconnected networks of functional units and associated proteins whose activity can be regulated by the application of internal and external stimuli. The majority of biological processes are controlled by chemical stimuli, such as ion concentrations and interactions with specific small-molecule effectors or inhibitors^1^. Molecular recognition, the ability of one molecule to ‘recognize’ another molecule through weak bonding interactions, is of fundamental importance to most processes within living systems^2^. The allosteric regulation of proteins by the binding of effector or inhibitor molecules at a site other than the active site is a powerful mechanism that drives complex biochemical reactions. Allosteric regulation is thus used to switch proteins from different conformational states to execute diverse functions^3^. The evolution of protein function is partially controlled by highly specific ligand binding sites that are crucial for the regulation of competing biological functions^4^. Furthermore, the reorganization of the chemical potential necessary for the action of a biochemical reaction can be attained by applying external stimuli. For example, changes in temperature can lead to the alteration of catalytic responses^5^, and the application of mechanical forces can induce chemical transformations^6^, among other reactions. Of the varied range of extrinsic stimuli, electromagnetic radiation is considered to be the most advantageous stimulus because it can precisely provide high spatio-temporal selectivity with strong dosage control^7^.

Nature has evolved photo-responsive proteins such as rhodopsin, which is regulated by the *cis*-*trans* isomerization of its cofactor retinal^8^. These photo-susceptible systems are generally composed of a photosensitive chromophore (photochromic molecule) that undergoes a chemical transformation (*e.g.*, photo-isomerization), subsequently controlling the conformation of the second functional unit (*e.g.*, a protein domain)^9^. A widely used approach to introduce a photochromic moiety is employed following the chemical modification of the protein^10^.

Two fundamental classes of photoswitchable biomolecules have been developed: single-cycle and multicycle photoswitches. In single-cycle photoswitches, biomaterial is deactivated by the attachment of photosensitive chemical-protecting groups. Multicycle photoswitches can control enzymatic activities and ligand-binding affinities through the direct photoisomerization of chromophores and *via* the interactions of low-molecular-weight photochromic compounds with biomaterials^11^. These photoswitches enable either irreversible or reversible control of biological activities through biomolecular recognition events.

Mono-functional or bi-functional azobenzene derivatives and spiropyrans have been used as photoswitches to modulate biological activities. Reversible transitions of these compounds are mediated either through *cis*-*trans* or syn-anti isomerization^12^. Additionally, azobenzene derivatives have been shown to chemically modify peptides and proteins^13^, altering the conformations and activities in a reversible manner in the presence of light^14^,^15^,^16^. In particular, photoswitchable proteins using azobenzene have been successfully employed to regulate protein function *via* light irradiation both *in vitro*^17^ and *in vivo*^18^. Moreover, enzymes have been modified with mono-functional^7^ or bi-functional azobenzene^9^ derivatives in such a way as to enable the modulation of catalytic activity by light^19^. The photo-control of enzymatic activity offers opportunities to develop novel biotechnological applications and is considered as a promising area of research in near future.

Herein, we report the photo-control of the enzymatic activity of the serine protease α-chymotrypsin (CHT) through a new class of photochromic material, dihydroindolizine (DHI)^20^,^21^,^22^. Photochromic DHI has received much attention owing to its remarkable photo-fatigue resistance and broad range of absorption^23^. Moreover, photochromic DHI is used to tether peptides and proteins *via* a maleimide functional group, which corresponds to absorption in the red region of the visible spectrum and in the near-IR spectral domain, indicating the potential for future use in *in vivo* applications^24^,^25^. The basis of the photochromic behavior of DHI is light-induced reversible pyrroline ring opening, which transforms the molecule from a light yellow colored form (*cis*) to a red colored betaine form (*trans*)^26^,^27^,^28^. Betaines undergo a thermal back reaction to their corresponding DHI *cis* form by 1,5-electrocyclization^22^,^29^. We have demonstrated that the recognition of photochromic DHI by CHT alters the enzymatic activity of CHT in a light-responsive manner. Circular dichroism (CD) spectra suggest that the protein structure remains unperturbed upon DHI recognition. To elucidate the position and orientation of the DHI moiety within CHT, Förster resonance energy transfer (FRET) strategy has been employed. 4-Nitrophenyl anthranilate (NPA), a covalently-attached chromophore at the active site of enzyme, has been used to obtain the distance between the active site and the DHI *cis*-isomer, which can then be correlated with the position of the DHI within CHT. In a similar manner, proflavin (PF) has been employed to measure the distance of the DHI *trans*-isomer from the CHT active site. Molecular docking has been utilized to specifically locate the position of both DHI isomers. The results of molecular docking correspond with our experimental findings. The molecular recognition of photochromic DHI by serine protease CHT leads to photo-responsive enzyme inhibition that could be used as a photo-controlled biological switch in numerous therapeutic applications.

## Results & Discussion

**Figure 1:**
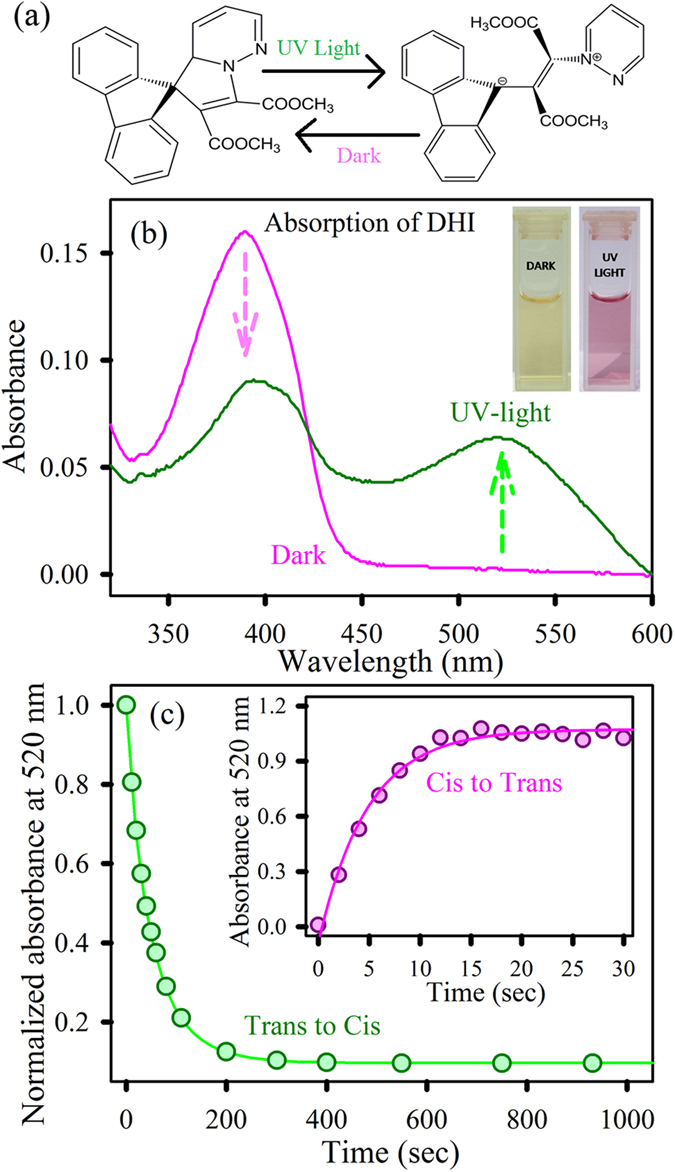
(**a**) Structures of the *cis* and *trans* isomers of DHI. (**b**) Absorption spectra of DHI: *cis* and *trans* isomers. The inset shows visible color change from yellow to dark pink owing to conversion. (**c**) Kinetics of the *trans* to *cis* conversion reaction of DHI in acetonitrile. Inset shows the corresponding *cis* to *trans* conversion rate.

To elucidate the molecular recognition of photochromic DHI by CHT, we optically characterized the ligand. Figure 1a shows the structures corresponding to the *cis*-isomer (present under dark conditions) and *trans*-isomer (present when the sample is exposed to UV-light) of the DHI ligand. In the presence of UV-light, ring opening of the *cis*-isomer *via* a cyclopropyl-allyl conversion leads to the formation of a colored *trans*-isomer (betaine). Subsequent slow thermal ring closure by 1,5-electrocyclization regenerates the *cis*-isomer^21^. The optical absorption spectrum of the *cis*-isomer shows a peak at 390 nm, which reduces in intensity when exposed to UV light, yielding a subsequent peak at 520 nm (Fig. 1b). These absorption bands can be assigned to the locally excited π-π* transition that occurs in the butadienyl-vinyl-amine chromophores. The inset demonstrates the visible color change of the sample from yellow to pink owing to the *cis* to *trans* conversion^23^. To evaluate the photochromic behavior of DHI, isomerization reaction kinetics are investigated in polar aprotic acetonitrile media. The *cis* to *trans* conversion is monitored by measuring the increase in absorbance at 520 nm. In a similar manner, the *trans* to *cis* conversion is followed by a decrease in absorbance at 520 nm. The rate constants for light triggered *cis* to *trans* conversion and *trans* to *cis* thermal relaxation have been depicted in Table 1.

**Figure 2:**
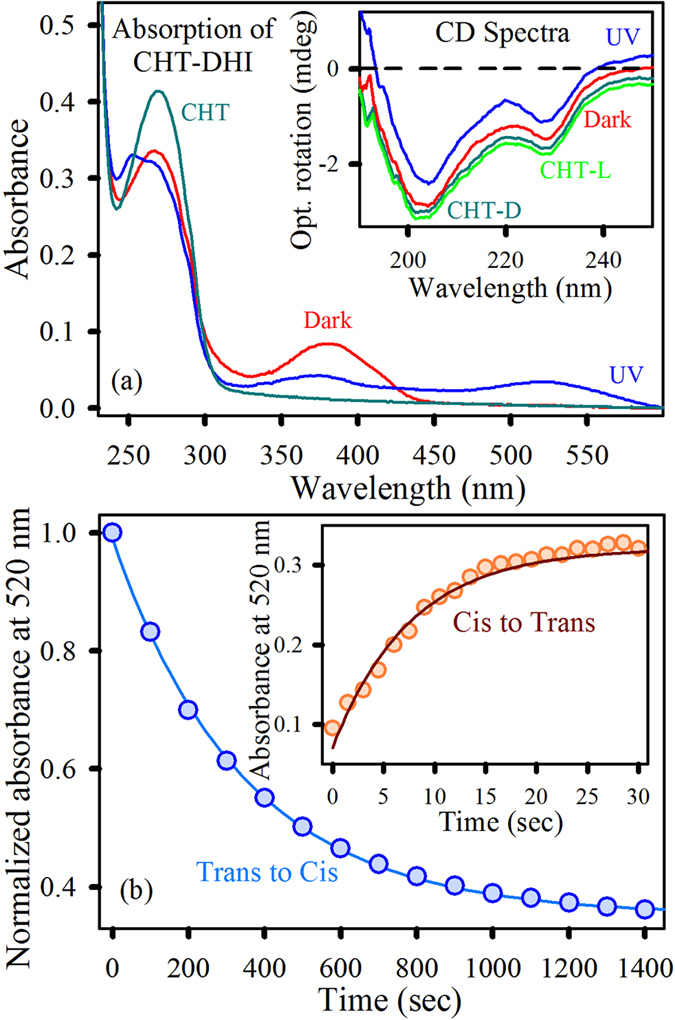
(**a**) Absorption spectra of CHT and CHT-DHI. The inset shows the corresponding CD spectra. (**b**) Kinetics of the trans to cis conversion reaction of CHT-DHI in phosphate buffer. Inset shows the corresponding cis to trans conversion rate.

After the DHI and CHT interaction, the absorption spectra of CHT-DHI has a peak at 390 nm, indicative of the incorporation of DHI into CHT. This peak reduces its intensity and generates a peak at 520 nm when exposed to UV light (Fig. 2a), which is characteristic of the photoisomerization of DHI to the betaine form. The absorption spectra clearly suggest a reasonable recognition of DHI (in both *cis* and *trans* isomers) by CHT. To confirm the structural integrity of the enzyme, we performed CD experiments of CHT and CHT-DHI (in dark and light conditions). Figure 2a (inset) shows the CD spectra of CHT. These spectra indicate two minima at 202 nm and 232 nm, corresponding to the native secondary and tertiary structures of the protein, respectively^30^. Upon UV illumination, the CD spectrum of CHT (green line in Fig. 2a inset) shows no alteration. There is no perturbation observed with respect to these two peaks in CHT-DHI, suggesting the structural integrity of the protein after interaction with photochromic DHI. The photoisomerization rate of DHI when confined in the protein environment is drastically reduced. The *cis* to *trans* conversion rate of CHT-DHI in phosphate buffer is found to be decreased compared to free-DHI conversion (inset of Fig. 2b). The quantum yield of *cis* to *trans* conversion is calculated to be 0.34 for free-DHI, whereas, for CHT-DHI, it is 0.1. The values are consistent with solvent dependent quantum yield of photochromic ligands, available in literatures^31^.The significant reduction in *cis* to *trans* isomerization can be rationalized by the fact that the hydrophobic DHI *cis*-isomer can fit into various available hydrophobic binding sites present in the CHT structure^32^. The structural stability of the DHI *cis*-isomer into CHT might be increased by hydrophobic interactions resulting in a decreased isomerization rate. Moreover, the *trans* to *cis* conversion rate is decreased further compared to free-DHI (almost 50 times less), as shown in Fig. 2b. In the case of the DHI *trans*-isomer, stability further increases owing to the presence of several non-covalent interactions of the ionic *trans*-isomer with the more polar solvent (water), which significantly reduces the rate of *trans* to *cis* conversion.

**Figure 3:**
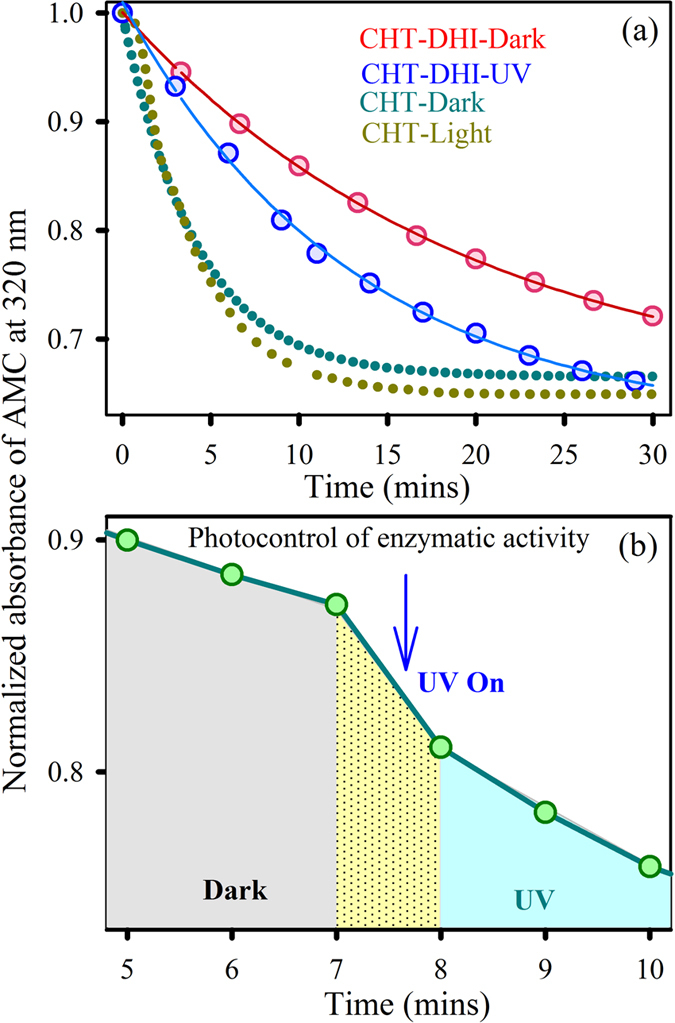
(**a**) Enzymatic activity of CHT-DHI in absence and presence of UV light. (**b**) Photo-control of enzymatic activity of CHT-DHI.

After confirming the successful interaction between CHT and DHI in the dark and in the presence of UV light, we explored the corresponding enzymatic activities. Under both conditions, the enzymatic activities are inhibited compared to free-CHT, as shown in Fig. 3a. Under dark conditions, the activity is more hindered compared to the light-irradiated condition, indicating that the *cis*-isomer is more capable of inhibiting CHT activity compared to the *trans*-isomer. To evaluate the photo-control of enzymatic activity, we performed enzyme catalysis reactions both in dark and light conditions for 7-minute intervals. Figure 3b clearly depicts the variation in reaction rate depending on the illumination condition. For the first 7 minutes in dark, the decay constant is much slower. However, for the next 7 minutes, the reaction rate increases in the presence of light (Table 1). Moreover, the photoresponsive enzymatic activity of CHT-DHI is recyclable for three consecutive cycles (data not shown) with a similar rate of reaction. This trend in enzymatic activity of CHT-DHI indicates that both the site of attachment and the conformational changes of DHI within the protein cavity are responsible for photo-controlled inhibition. Hence, the specific position of recognition and orientation of DHI in the protein cavity are two important parameters for an in-depth investigation of the molecular interactions.

**Figure 4:**
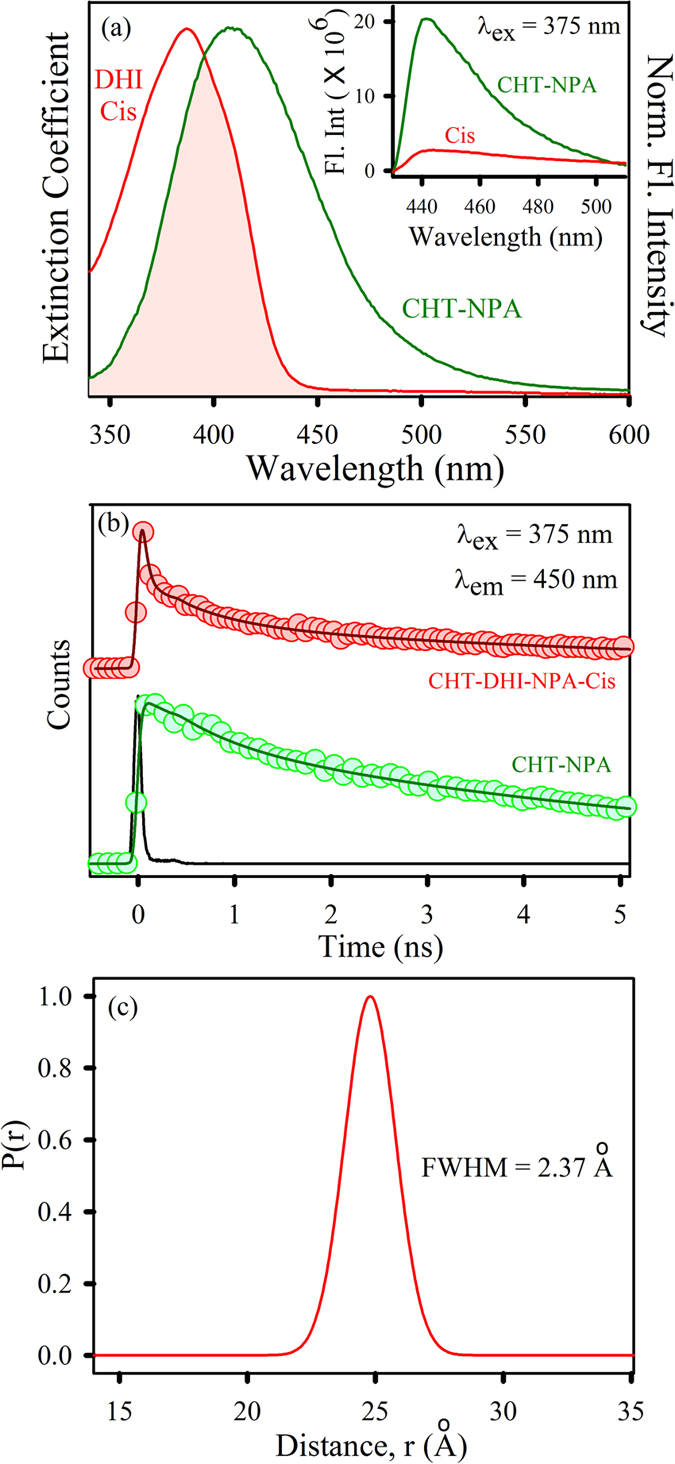
(**a**) Overlap of the CHT-NPA emission and DHI *cis* absorption. Inset shows PL spectra of CHT-NPA and CHT-DHI-NPA upon excitation at 375 nm. (**b**) The fluorescence transients of CHT-NPA (excitation at 375 nm) in the absence and in the presence of DHI *cis* collected at 450 nm are shown. (**c**) Distribution of donor-acceptor distances between CHT-NPA and the DHI *cis-*isomer.

**Figure 5:**
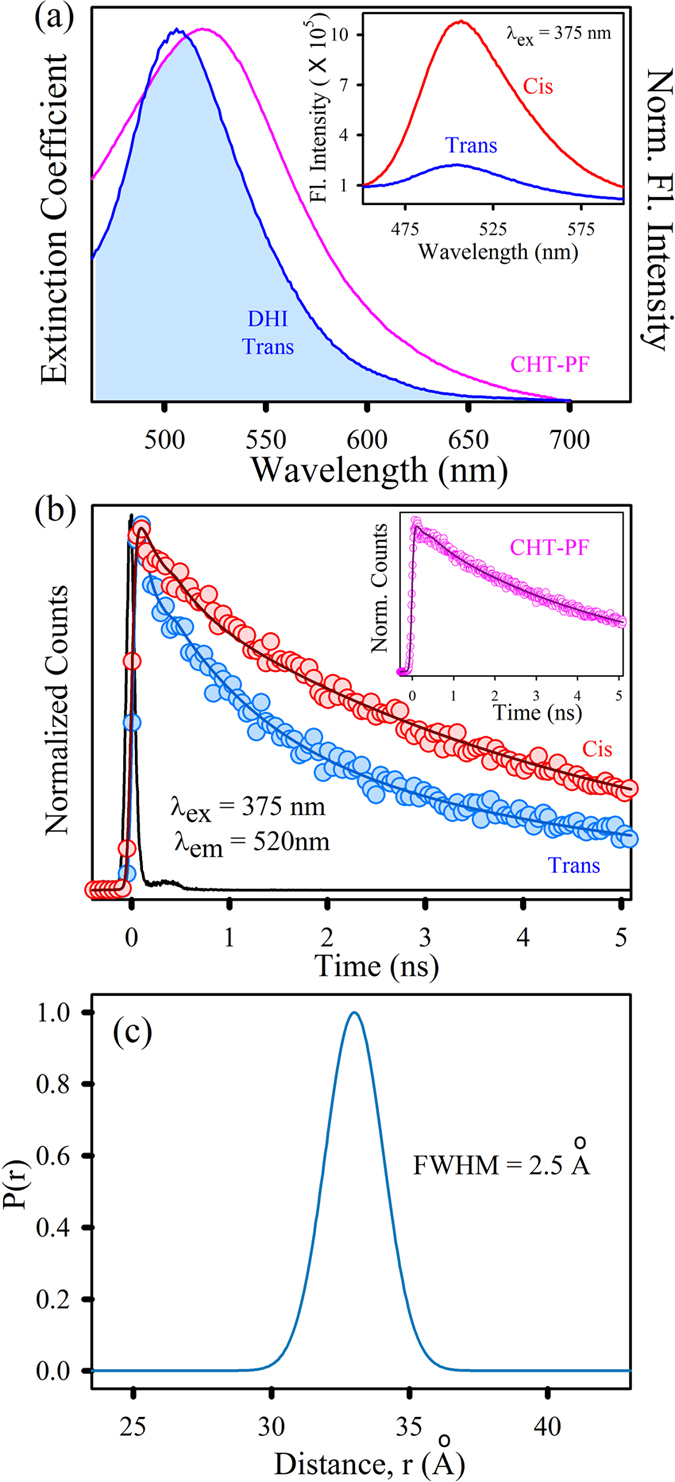
(**a**) The overlap of the CHT-PF emission and DHI *trans* absorption. Inset shows the PL spectra of CHT-DHI-PF *cis* and CHT-DHI-PF *trans* upon excitation at 375 nm. (**b**) The fluorescence transients of CHT-DHI-PF (excitation at 375 nm) in *cis* and in *trans* forms collected at 520 nm are shown. Inset depicts the fluorescence transients of CHT-PF. (**c**) Indicates the distribution of donor-acceptor distances between the CHT-PF and DHI (*trans*).

Considering the hydrophobic nature of DHI, the possibility of interaction with the S1 pocket (active site) of CHT is more likely to take place and was hence investigated by the addition of the well-established fluorescent probes NPA and PF separately to the CHT-DHI complex. The simultaneous binding of DHI and either NPA or PF to CHT offers a unique opportunity to use Förster resonance energy transfer (FRET) as a tool to measure the relative position of the two probes one of which acting as a donor and the other as acceptor within the protein cavity. In this case, CHT-NPA or CHT-PF is acting as the donor in the protein cavity and DHI acts as the acceptor. Figure 4a shows a spectral overlap between CHT-NPA emission, and the absorption of the DHI *cis*-isomer suggests a possibility of FRET from CHT-NPA to DHI. The significant decrease in the steady-state emission of CHT-NPA upon DHI *cis*-isomer attachment is shown in the inset of Fig. 4a. The picosecond-resolved fluorescence decay profile of the donor CHT-NPA in the presence and absence of the acceptor DHI *cis*-isomer is obtained upon excitation with a 375-nm laser and is monitored at 450 nm (Fig. 4b). A shorter excited state lifetime of the CHT-NPA is clearly observed in the presence of the DHI *cis*-isomer. Details of the spectroscopic fitting parameters of the fluorescence transients are tabulated in Table 2. From FRET calculations, the distance between the donor CHT-NPA and acceptor DHI *cis*-isomer are determined to be 2.5 nm, and the energy transfer efficiency is calculated to be 79%. This observation confirms that the DHI *cis*-isomer attachment site is located at a distance of 2.5 nm from the NPA binding site. As documented in the literature, NPA binds at the hydrophobic active site of CHT^33^, and it has been subsequently proven that the DHI *cis*-isomer binds at a site other than the S1 pocket. The distribution of donor–acceptor distances in the CHT-DHI (*cis)*-NPA (Fig. 4c) reveals an internal fluctuation with a full-width half-maximum (FWHM) of 2.37 Å. The relative position of the DHI *cis*-isomer is located at a distance of 25 ± 2.37 Å from the active site of CHT. The absorption spectra of the DHI *trans*-isomer overlaps with the emission of CHT-NPA and shows a faster excited state lifetime with an energy transfer efficiency of 74% (data not shown). However, to confirm the sole contribution of the *trans*-isomer, we employed the simultaneous binding of PF and DHI in CHT, enabling the possibility of energy transfer from CHT-PF (donor) to the DHI *trans-*isomer (*i.e.*, when illuminated with UV light). The spectral overlap of the donor CHT-PF emission with the DHI *trans*-isomer absorption spectrum is shown in Fig. 5a. The steady-state fluorescence quenching of CHT-DHI(*trans*)-PF compared to CHT-DHI(*cis*)-PF is evident (Fig. 5a). The fluorescence decay profile of the donor CHT-PF in the absence of the acceptor DHI (Fig. 5b inset) and in the presence of the acceptor DHI in both isomers (Fig. 5b) are obtained upon excitation with a 375 nm laser and monitoring at 520 nm. The excited state lifetime of the CHT-PF quenches in the CHT-DHI(*trans*)-PF, indicating the presence of an energy transfer process. The details of the spectroscopic parameters and the fitting parameters of the fluorescence decays can be found in Table 2. From FRET calculations, the distance between the donor CHT-PF and acceptor DHI trans-isomer is determined to be 3.3 nm. The energy transfer efficiency is calculated to be 61%. Figure 5c depicts the distribution of the donor–acceptor distances in the CHT-DHI(*trans*)-PF, revealing an internal fluctuation with a full-width half-maximum (FWHM) of 2.5 Å. PF is also known to be an active site binding inhibitor^34^ of CHT, and it can be suggested that the relative position of the DHI *trans*-isomer is at a distance of 33 ± 2.5 Å from the CHT active site. Because enzymatic inhibition does not result from competition with the substrate at the Ser-195-His-57 active site, inhibition must result from other interactions with a control or allosteric site. This allosteric site may be hydrophobic in nature, and thus the hydrophobic inhibitor may bind *via* electrostatic and hydrophobic interactions. These interactions result in an unfavorable conformational change at some different sites on the enzyme, significantly hampering its enzymatic activity^35^.

**Figure 6:**
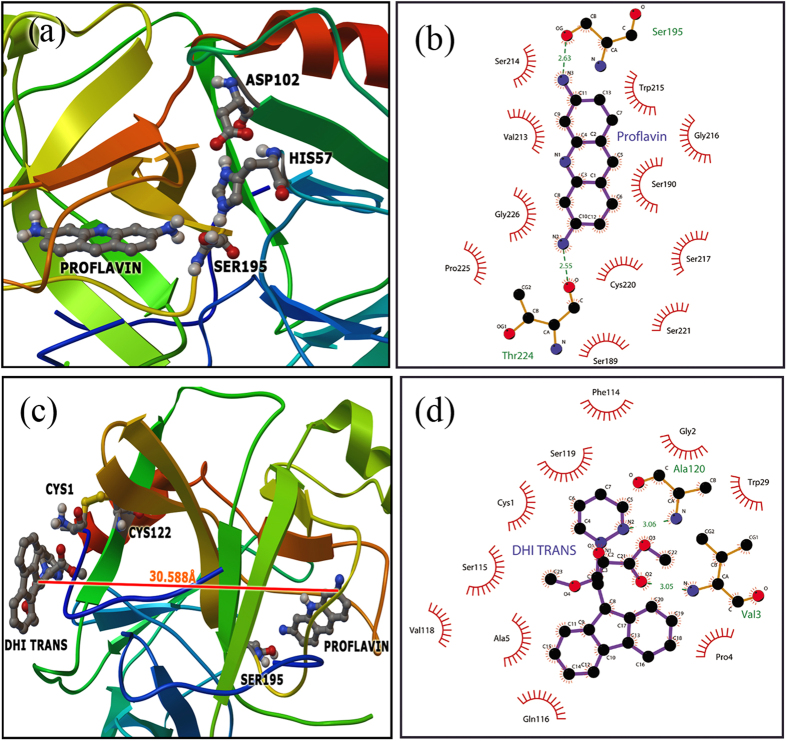
(**a**) Relative position of PF in CHT cavity. (**b**) Molecular contacts between the amino acid residues of CHT and PF. (**c**) Relative position of the DHI *trans*-isomer in the CHT-PF cavity. (**d**) Molecular contacts between the amino acid residues of CHT-PF and the DHI *trans*-isomer.

**Figure 7:**
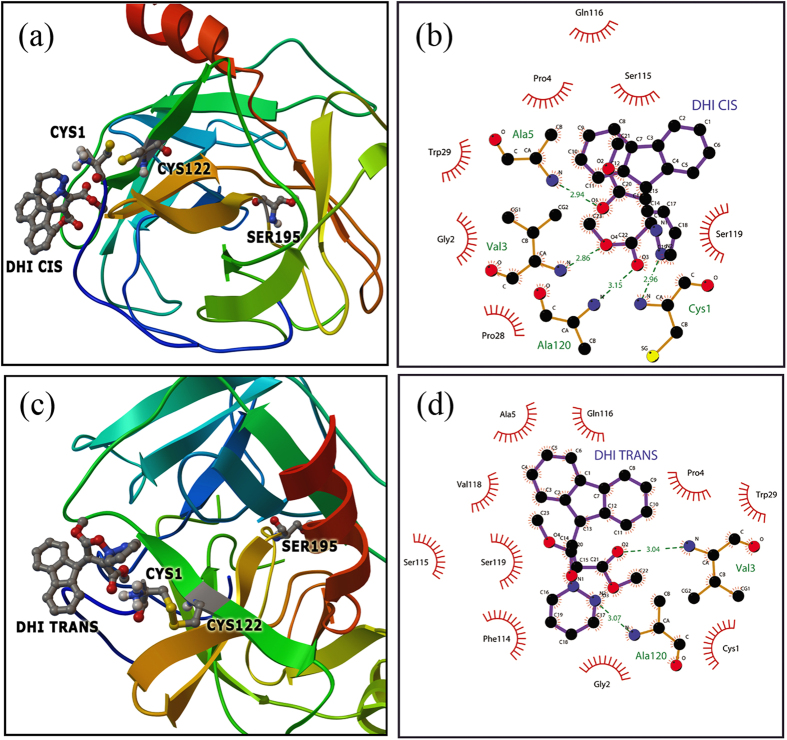
(**a**) Relative position of the DHI *cis*-isomer in the CHT cavity. (**b**) Molecular contacts between the amino acid residues of CHT and DHI *cis*-isomer. (**c**) Relative position of the DHI *trans*-isomer in the CHT cavity. (**d**) Molecular contacts between the amino acid residues of CHT and DHI *trans*-isomer.

To investigate the specific site of interactions between DHI and CHT, flexible docking studies were performed. We first employed PF as the ligand and CHT as the macromolecule. The most likely ligand binding modes are obtained by extracting the lowest energy conformation from the largest cluster (Fig. 6a). The flexible docking results clearly suggest that PF binds near the catalytic triad. Moreover, within the flexible docking binding sites of PF, there are possible hydrogen bond interactions between the N3 of PF and Ser-195 (2.63 Å) and between the N2 of PF and Thr 224 (2.55 Å) (Fig. 6b). Additionally, Trp-215, Ser-190, Gly-226, Val-213 and Cys-220 could provide additional hydrophobic force to stabilize the complex (Fig. 6b). The lowest energy structure obtained from CHT-PF in a blind docking calculation is considered to be the macromolecule for evaluating the site of DHI interaction. Accordingly, the DHI *trans-*isomer is considered to be the flexible ligand and CHT-PF, the macromolecule. The lowest energy conformer occupies a site near the Cys-1-Cys-122 ANS binding hydrophobic site. The distance between the base of PF and DHI is measured to be 30.5 Å (Fig. 6c), which is consistent with our experimental findings. There are possible hydrogen bond interactions between the N2 of DHI and Ala-120 (3.06 Å) and between the O2 of DHI and Val-3 (3.05 Å). Additionally, Ser-119, Cys-1, Ala-5 and Gln-116 residues could provide extra hydrophobic force to stabilize the complex and several electrostatic interactions are also present. Finally, flexible docking using both DHI isomers separately as the ligand and CHT as the macromolecule suggests that both isomers of DHI are bound to the same site in CHT (near the hydrophobic Cys-1-Cys-122). However, the difference in orientation and possible various interactions leads to dissimilarity with respect to enzymatic activities. There are four possible hydrogen bond interactions between the N2 of DHI and Cys-1 (2.96 Å), between the O4 of DHI and Val-3 (2.86 Å), between the O1 of DHI and Ala-5 (2.94 Å) and between the O3 of DHI and Ala-120 (3.15 Å) for the DHI *cis*-isomer, whereas the *trans*-isomer is able to form two hydrogen bonds between the O2 of DHI and Val-3 (3.04 Å) and between the N2 of DHI and Ala-120 (3.07 Å) (Fig. 7). The corresponding electrostatic and hydrophobic interactions with various amino acid residues are also distinct in the case of both DHI isomers. The hydrophobic attachment of a molecule to a control site of an enzyme accompanying non-covalent interactions develops an unfavorable conformation, leading to a less effective active site for enzymatic action. In this study, the *cis*-isomer is more hydrophobic and interacts with the protein cavity to a greater extent compared to the *trans*-isomer. Therefore, a higher perturbation occurs with regard to its enzymatic activity (allosteric inhibition) compared to the *trans*-isomer, which is consistent with our experimental findings. Thus, the diversity in molecular recognition of a photochromic ligand under dark and light conditions leads to the photo-control of enzymatic action.

## Conclusion

In this study, we investigated the molecular basis of the photo-control of serine protease α-chymotrypsin (CHT) enzymatic activity *via* the incorporation of a new class of synthesized photochromic material, dihydroindolizine (DHI). Photo-fatigue resistance DHI exhibits a broad range of absorption and possesses a variety of prospective applications. The light-induced reversible pyrroline ring opening and the thermal back recovery reaction are responsible for photochromism. We have demonstrated that the recognition of photochromic DHI by CHT inhibits enzymatic activity in a light-responsive manner. Circular dichroism studies suggest no perturbation in the secondary structure of the enzyme structure upon interaction with DHI. Moreover, steady-state absorption studies suggest that DHI interacts with the protein cavity. To elucidate the position and orientation of the DHI moiety within CHT under dark and UV-illuminated conditions, we applied Förster resonance energy transfer (FRET). A covalently attached chromophore at the active site of the enzyme 4-nitrophenyl anthranilate (NPA) was employed to obtain the distance between the active site and the DHI *cis*-isomer. A distance of 25 Å revealed the possible location of the DHI *cis*-isomer at an additional hydrophobic site rather than the catalytic center. In a similar manner, using the probe proflavin (PF), the distance of the DHI *trans*-isomer from the active site of CHT was estimated to be 33 Å. Finally, using molecular docking simulations, the locations of both DHI isomers were identified to be near the hydrophobic Cys-1-Cys-122 region. The relative orientations and variations in the electrostatic or hydrophobic interactions for both DHI isomers within the protein cavity are responsible for altering enzymatic activity. Thus, the diversity of the molecular recognitions of a photochromic ligand leads to photo-controlled enzymatic modulation, which could be relevant as a photo-controlled biological switch in the near future.

## Experimental Section

### Materials

Bovine pancreatic α-chymotrypsin (CHT), Ala-Ala-Phe- 7-amido-4-methylcoumarin (AMC), 4-nitrophenylanthranilate (NPA) and proflavin (PF) were obtained from Sigma. The solutions were prepared in 100 mM phosphate buffer (pH 7.0) using water from Millipore. Acetonitrile and dichloromethane of spectroscopic grade were used as solvents.

### Synthesis of photochromic DHI

The photochromic DHI was synthesized^20^ by the electrophilic addition of electron-deficient spirocyclopropenes through the nitrogen atom of the N-heterocyclic pyridazines in dry ether in the absence of light under nitrogen atmosphere for 24 h. The final photochromic DHI was obtained as pale yellow crystals after recrystallization from the proper solvent. Pure products were obtained after purification by column chromatography on silica gel using dichloromethane as eluent^23^.

### Preparation of CHT-DHI solution

Enzyme-DHI solutions were prepared by adding a requisite amount of DHI to the enzyme solution with stirring for 2 h. To ensure complete complexation of DHI with the enzyme, the DHI concentration was kept much lower (20 μM) than the CHT concentration (100 μM). For energy transfer studies, the probes (NPA and PF; 20 μM concentration) were added to the CHT-DHI solution and stirred for 2 h. For the enzymatic activity studies, the enzyme concentration was 5 μM in all cases, and the concentration of the substrate AMC was 125 μM. Additional details about enzymatic studies can be found elsewhere^36^.

### Characterization Techniques

Steady-state absorption and emission were measured with a Shimadzu UV-2600 spectrophotometer and Jobin Yvon Fluorolog fluorimeter, respectively. CD spectra were recorded using a JASCO-810 spectropolarimeter. All kinetics measurements of the isomerization reaction were performed with the SPECTRA SUITE software supplied by Ocean Optics. All picosecond resolved fluorescence transients were measured using a commercially available time-correlated single-photon counting (TCSPC)^37^ setup with MCP-PMT from Edinburgh Instruments, U.K. (instrument response function (IRF) of ~70 ps) using a 375 nm excitation laser source. To estimate the FRET efficiency of the energy donor (D) to the acceptors (A) and hence to determine the distance of the FRET pair (D–A), we followed the same methodology described in earlier reports^38^,^39^,^40^. The distance distribution function, P(r), was calculated using the nonlinear least-squares fitting procedure using SCIENTIST^TM^ software and following a Gaussian function with a standard deviation of σ and r as the donor-acceptor distance. Detailed theory and calculation of the distance distribution can be found elsewhere^41^,^42^. A UV light source (LED) of 390 nm – 400 nm was used to isomerize the DHI solution To compare photo-isomerization reactions, all the external parameters such as–lamp power, UV irradiation time, temperature and solvent are kept same.

### Details of photo-isomerization reaction

To measure the photo-isomerization rate of light triggered *cis* to *trans* conversion, we have measured the absorbance of the samples while irradiating with UV light source (LED). The temperature is maintained at 25 °C. For measuring the thermal relaxation process, we have irradiated the samples with the same UV lamp source for 7 mins to ensure complete conversion from *cis* to *trans*. After switching off the lamp, we measured the absorbance of the solutions. We have used SPECTRA SUITE software supplied by Ocean Optics to measure the absorbance values. Moreover, the time interval between measurements of two consecutive absorbance values is kept at 50 ms. The *cis* to *trans* conversion is monitored by measuring the increase in absorbance at 520 nm. In a similar manner, the trans to cis conversion is followed by a decrease in absorbance at 520 nm.

### Details of molecular docking calculations

The crystal structure of CHT was obtained from the Protein Data Bank (entry code 1YPH). The ligand files were prepared and optimized using the Avogadro software package. All molecular docking simulations of CHT-DHI (*cis* and *trans*) were performed with the AutoDock Tools-1.5.6 program^43^. Blind grid computation was performed with a grid box of 126 × 126 × 126 A with 0.375 Å spacing, covering all of the active site residues and allowing rotatable ligand bond flexibility with 10 GA runs, and finally, terminating after a maximum of 25 × 10^6^ energy evaluations. The population size was set to 150 with a crossover rate of 0.8 (Lamarckian Genetic Algorithm). For the site-specific flexible docking, a grid box of 58 × 62 × 64 A with 0.375 Å spacing covering the lowest energy ligand binding site obtained in the blind docking calculation was used, and all other parameters were the same as the earlier blind docking case. The lowest energy conformation in the largest cluster of each docking simulation was extracted and analyzed. The hydrogen bonds and hydrophobic interactions between the ligands and the protein were visualized with Ligplot^+^ version 1.4.5. The Ligplot images show hydrogen bond interaction in green and hydrophobic interactions in red.

## Additional Information

**How to cite this article**: Bagchi, D. *et al.* Allosteric Inhibitory Molecular Recognition of a Photochromic Dye by a Digestive Enzyme: Dihydroindolizine makes α-chymotrypsin Photo-responsive. *Sci. Rep.* **6**, 34399; doi: 10.1038/srep34399 (2016).

## Figures and Tables

**Table 1:** Time constants of the isomerization reaction and enzymatic activity of CHT-DHI.

Isomerization Reaction	Systems	Time Constant (sec)	
		DHI (in acetonitrile)	CHT-DHI (in buffer)
	*Cis* to *trans*	4.8	7.6
	*Trans* to *cis*	53.3	342.9
Enzymatic Activity		**Time Constant (min)**	
		**Dark**	**Light**
	CHT	4.0	4.0
	CHT-DHI	100	5.0
		D1	L1
	Dark then Light	100	4.8

The same lamp source is used for all the light triggered processes. Moreover, the temperature is maintained at 25 °C.

**Table 2:** Picosecond-resolved fluorescence transient lifetime.

Fluorescence transient lifetime	System	τ_1_ (ns)	τ_2_ (ns)	τ_3_ (ns)	τ_avg_ (ns)
	CHT-NPA		0.6 (29.6%)	5.9 (70.4%)	4.4
	CHT-DHI-NPA-*Cis*	0.04 (71.5%)	0.6 (14.7%)	5.7 (13.8%)	0.9
	CHT-DHI-NPA-*Trans*	0.07 (51%)	0.6 (31%)	5.1 (18%)	1.2
	CHT-PF		0.4 (16%)	4.8 (84%)	4.1
	CHT-DHI-PF-*Cis*		0.3 (29.1%)	4.5 (70.9%)	3.3
	CHT-DHI-PF-*Trans*	0.067 (40.5%)	1.0 (32.4%)	4.6 (27.1%)	1.6
FRET parameters		**J (λ)**	**R**_**0**_**(Å)**	**Efficiency**	**r**_**DA**_ **(nm)**
	CHT-DHI-NPA-*Cis*	1.426 × 10^14^	31.1	79.3	2.5
	CHT-DHI-PF-*Trans*	0.488 × 10^15^	35.56	61.2	3.3

The emission (monitored at 450 nm for NPA systems and at 520 nm for PF systems) was detected using an excitation laser at 375 nm. Numbers in parentheses indicate relative contributions. The reported lifetimes carry ~5% uncertainties.
